# Comparing different deep learning architectures for classification of chest radiographs

**DOI:** 10.1038/s41598-020-70479-z

**Published:** 2020-08-12

**Authors:** Keno K. Bressem, Lisa C. Adams, Christoph Erxleben, Bernd Hamm, Stefan M. Niehues, Janis L. Vahldiek

**Affiliations:** 1 Charité Universitätsmedizin Berlin, Campus Benjamin Franklin, Hindenburgdamm 30, 12203 Berlin, Germany; 2 Charité Universitätsmedizin Berlin, Campus Mitte, Charitéplatz 1, 10117 Berlin, Germany

**Keywords:** Medical research, Radiography

## Abstract

Chest radiographs are among the most frequently acquired images in radiology and are often the subject of computer vision research. However, most of the models used to classify chest radiographs are derived from openly available deep neural networks, trained on large image datasets. These datasets differ from chest radiographs in that they are mostly color images and have substantially more labels. Therefore, very deep convolutional neural networks (CNN) designed for ImageNet and often representing more complex relationships, might not be required for the comparably simpler task of classifying medical image data. Sixteen different architectures of CNN were compared regarding the classification performance on two openly available datasets, the CheXpert and COVID-19 Image Data Collection. Areas under the receiver operating characteristics curves (AUROC) between 0.83 and 0.89 could be achieved on the CheXpert dataset. On the COVID-19 Image Data Collection, all models showed an excellent ability to detect COVID-19 and non-COVID pneumonia with AUROC values between 0.983 and 0.998. It could be observed, that more shallow networks may achieve results comparable to their deeper and more complex counterparts with shorter training times, enabling classification performances on medical image data close to the state-of-the-art methods even when using limited hardware.

## Introduction

Chest radiographs are among the most frequently used imaging procedures in radiology. They have been widely employed in the field of computer vision, as chest radiographs are a standardized technique and, if compared to other radiological examinations such as computed tomography or magnetic resonance imaging, contain a smaller group of relevant pathologies. Although many artificial neural networks for the classification of chest radiographs have been developed, it is still subject to intensive research.

Only a few groups design their own networks from scratch, while most use already established architectures, such as ResNet-50 or DenseNet-121 (with 50 and 121 representing the number of layers within the respective neural network)^1–6^. These neural networks have often been trained on large, openly available datasets, such as ImageNet, and are therefore already able to recognize numerous image features. When training a model for a new task, such as the classification of chest radiographs, the use of pre-trained networks may improve the training speed and accuracy of the new model, since important image features that have already been learned can be transferred to the new task and do not have to be learned again. However, the feature space of freely available data sets such as ImageNet differs from chest radiographs as they contain color images and more categories. The ImageNet Challenge includes 1,000 possible categories per image, while CheXpert, a large freely available data set of chest radiographs, only distinguishes between 14 categories (or classes)^7^, and the COVID-19 Image Data Collection only differentiates between three classes^8^. Although the ImageNet challenge showed a trend towards higher accuracies through increasing the number of layers in a CNN, this may not be necessary for a medical image classification task.

In radiology, sometimes only limited features of an image can be decisive for the diagnosis. Therefore, images cannot be scaled down as much as desired, as the required information would otherwise be lost. But the more complex a CNN, the more resources are required for training and deployment. As up-scaling the input-images resolution exponentially increases memory usage during training for large neural networks that evaluate many parameters, the size of a mini batch needs to be reduced earlier and more strongly (in ranges between 2 and 16), which may affect optimizers such as the stochastic gradient descent.

Therefore, it remains to be determined, which of the available artificial neural networks designed for and trained on the ImageNet dataset will perform best for the classification of chest radiographs. The hypothesis of this work is that the number of layers in a CNN is not necessarily decisive for good performance on medical data. CNN with fewer layers might perform similarly to deeper/more complex networks, while at the same time requiring less resources. Therefore, we systematically investigate the performance of sixteen openly available CNN on the CheXpert dataset and the COVID-19 Image Data Collection.

## Methods

### Data preparation

The free available CheXpert dataset consists of 224,316 chest radiographs from 65,240 patients. Fourteen findings have been annotated for each image: enlarged cardiomediastinum, cardiomegaly, lung opacity, lung lesion, edema, consolidation, pneumonia, atelectasis, pneumothorax, pleural effusion, pleural other, fracture and support devices. Hereby the findings can be annotated as present (1), absent (NA) or uncertain (− 1). Similar to previous work on the classification of the CheXpert dataset^3,9^, we trained these networks on a subset of labels: cardiomegaly, edema, consolidation, atelectasis and pleural effusion. As we only aim at network comparison and not on maximal precision of a neural network, for this analysis, each image with an uncertainty label was excluded, and other approaches such as zero imputation or self-training were also not adopted. Furthermore, only frontal radiographs were used, leaving 135,494 images from 53,388 patients for training. CheXpert offers an additional dataset with 235 images (201 images after excluding uncertainty labels and lateral radiographs), annotated by two independent radiologists, which is intended as an evaluation dataset and was therefore used for this purpose.

The COVID-19 Image Data Collection is a dataset focusing on chest radiographs for the novel coronavirus SARS-CoV-2 with the associated COVID-19 pneumonia. The dataset is still under active development, at the time of our analysis it consists out of 46,754 chest radiographs, of which 30,174 represent normal cases without pneumonia, 16,384 are cases with non-COVID-19 pneumonia and 196 include radiographs of confirmed COVID-19 pneumonia. We split the set into a dataset for training and validation consisting of 43,754 cases (28,240 normal, 15,333 non-COVID-19 pneumonia and 181 COVID-19) and a dataset for testing consisting including 3,000 cases (1,934 normal, 1,051 non-COVID-19 pneumonia and 15 COVID-19).

### Data augmentation

For the first and second training session, the images were scaled to 320 × 320 pixels, using bilinear interpolation, and pixel values were normalized. During training, multiple image transformations were applied: flipping of the images alongside the horizontal and vertical axis, rotation of up to 10°, zooming of up to 110%, adding of random lightning or symmetric wrapping.

### Model training

15 different convolutional neural networks (CNN) of five different architectures (ResNet, DenseNet, VGG, SqueezeNet, Inception v4 and AlexNet) were trained on two datasets^1,2,10–13^. All training was done using the Python programming language (https://www.python.org, version 3.8) with the PyTorch (https://pytorch.org) and FastAI (https://fast.ai) libraries on a workstation running on Ubuntu 18.04 with two Nvidia GeForce RTX 2080ti graphic cards (11 GB of RAM each)^14,15^. In the first training session, batch size was held constant at 16 for all models, while it was increased to 32 for all networks in the second session. We decided to use two different batch sizes, because maximum batch size is limited mainly by the available GPU-RAM and therefore can only increase to a limited amount, especially in larger and thus more memory-demanding networks. Especially with increased image resolution, lowering the batch size will be the major limitation to network performance.

Each model was trained for eight epochs, whereas during the first five epochs only the classification-head of each network was trained. Thereafter, the model was unfrozen and trained as whole for three additional epochs. Before training and after the first five epochs, the optimal learning rate was determined^16^. For CheXpert, it was between 1e − 1 and 1e − 2 for the first five epochs and between 1e − 5 and 1e − 6 for the rest of the training, while for the COVID-19 Image Data Collection, it was between 1e − 2 and 1e − 3 for the first five epochs and 1e − 5 and 1e − 6 for the rest of the training. We trained one multilabel classification head for each model for the CheXpert dataset and a multi-class model for the COVID-19 Image Data Collection. Since the performance of a neural network can be subject to minor random fluctuations, the training was repeated for a total of five times. The predictions on the validation data set were then exported as comma separated values (CSV) for evaluation.

### Evaluation

Evaluation was performed using the “R” statistical environment including the “tidyverse” and “ROCR” libraries^17–19^. Predictions on the validation dataset of the five models for each network architecture were pooled so that the models could be evaluated as a consortium. For each individual prediction as well as the pooled predictions, receiver operation characteristic (ROC) curves and precision recall curves (PRC) were plotted and the areas under each curve were calculated (AUROC and AUPRC). AUROC and AUPRC were chosen as they enable a comparison of different models, independent of a chosen threshold for the classification. Sensitivity and specificity were calculated with an individual cut-off for each network. The cut-off was chosen so that the sum of sensitivity and specificity was the highest achievable for the respective network.

## Results

The CheXpert validation dataset consists of 234 studies from 200 patients, not used for training with no uncertainty-labels. After excluding lateral radiographs (n = 32), 202 images from 200 patients remained. The dataset presents class imbalances (% positives for each finding: cardiomegaly 33%, edema 21%, consolidation 16%, atelectasis 37%, pleural effusion 32%), so that the AUPRC as well as the AUROC are reported. The performance of the tested networks is compared to the AUROC reported by Irvin et al.^3^ However, only values for AUROC, but not for AUPRC, are provided there.

In most cases, the best results were achieved with a batch size of 32, so all the information provided below refers to models trained with this batch size. Results achieved with smaller batch sizes of 16 will be explicitly mentioned.

### Area Under the Receiver Operating Characteristic Curve

**Figure 1:**
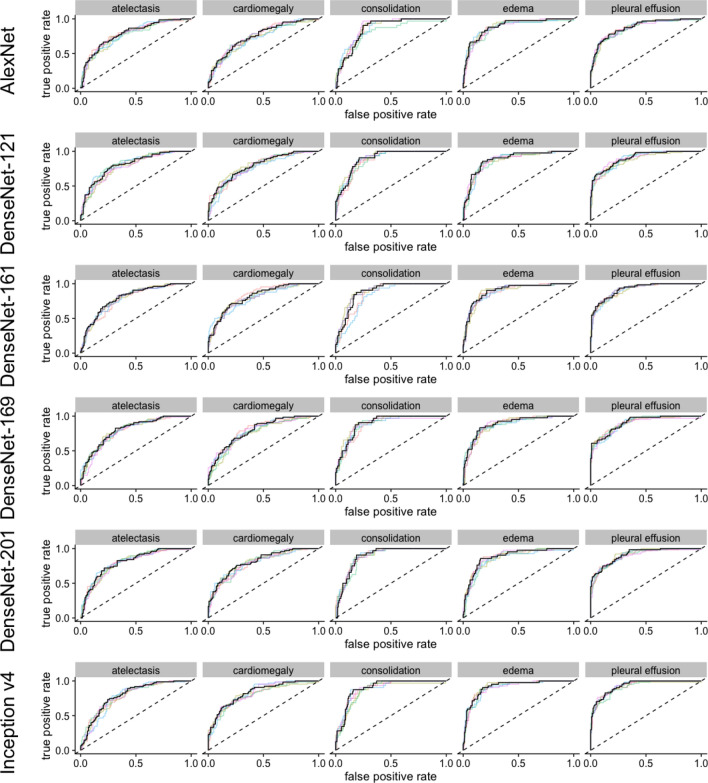
ROC curves for AlexNet, DenseNet and Inception v4 models. The colored lines represent a single training, black lines represent the pooled performance over five trainings. The figure was generated in R^20^.

**Figure 2:**
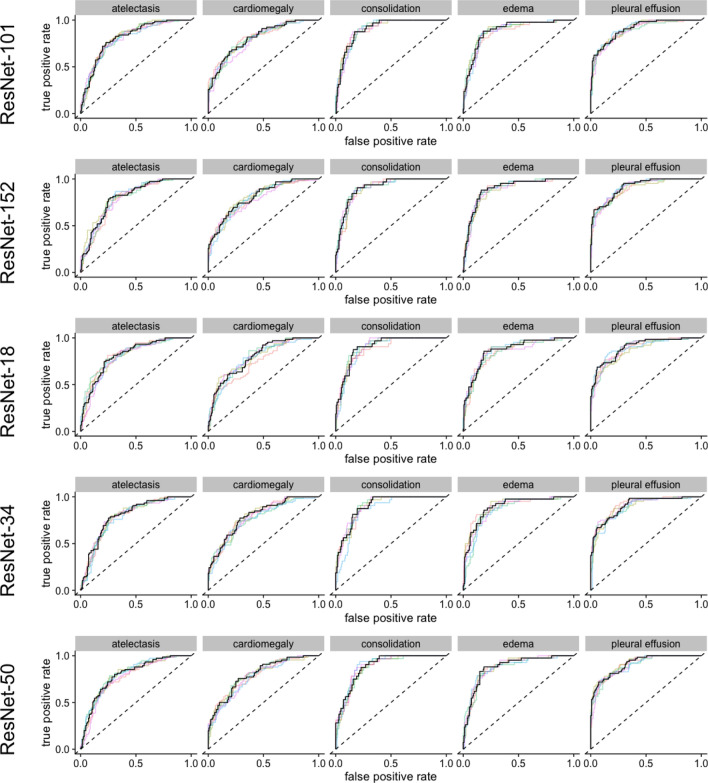
ROC-curves for the models with ResNet architectures. The colored lines represent a single training, black lines represent the pooled performance over five trainings. The figure was generated in R^20^.

**Figure 3:**
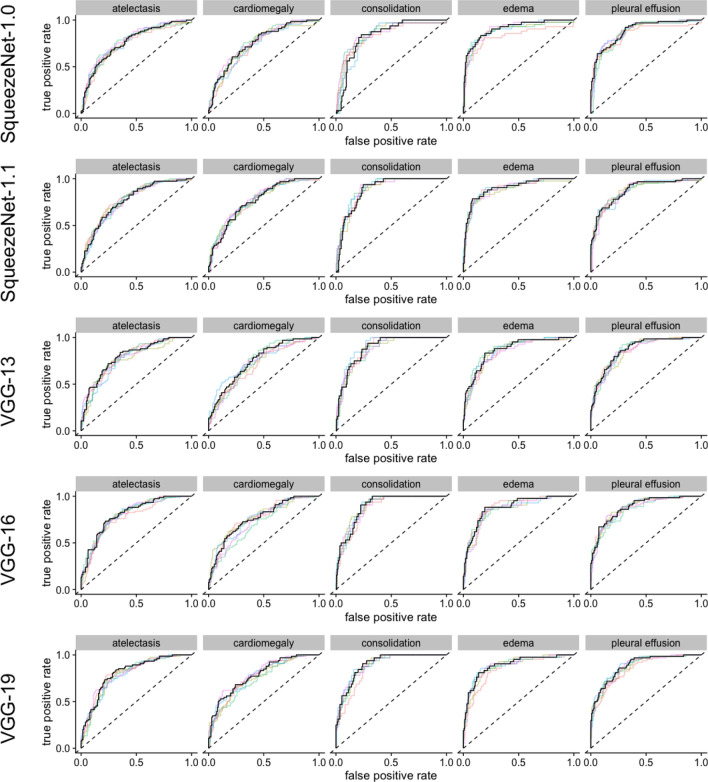
ROC-curves for the models with Squeezenet and VGG architectures. The colored lines represent a single training, black lines represent the pooled performance over five trainings.

On the CheXpert dataset, deeper CCN generally achieved higher AUROC values than shallow networks (Table 1 and Figs. 1, 2, 3). Regarding the pooled AUROC for the detection of the five pathologies, ResNet-152 (0.882), DenseNet-161 (0.881) and ResNet-50 (0.881) performed best (Irvin et al. CheXpert baseline 0.889)^3^. Broken down for individual findings, the most accurate detection of atelectasis was achieved by ResNet-18 (0.816, batch size 16), ResNet-101 (0.813, batch size 16), VGG-19 (0.813, batch size 16) and ResNet-50 (0.811). For detection of cardiomegaly, the best four models surpassed the CheXpert baseline of 0.828 (ResNet-34 0.840, ResNet-152 0.836, DenseNet-161 0.834, ResNet-50 0.832). For congestion, the highest AUROC was achieved using ResNet-152 (0.917), ResNet-50 (0.916) and DenseNet-161 (0.913). Pulmonary edema was most accurately detected using DenseNet-161 (0.923), DenseNet-169 (0.922) and DenseNet-201 (0.922). For pleural effusion, the four best models were ResNet-152 (0.937), ResNet-101 (0.936), ResNet-50 (0.934) and DenseNet-169 (0.934), all of which performed superior to the CheXpert baseline of 0.928.

**Table 1:** Area under the receiver operating characteristic curves—CheXpert.

Network	Batchsize	Atelectasis	Cardiomegaly	Consolidation	Edema	Effusion	Pooled
CheXpert baseline	16	0.818	0.828	0.938	0.934	0.928	0.889
AlexNet	16	0.790	0.755	0.857	0.894	0.881	0.835
DenseNet-121	16	0.809	0.794	0.895	0.883	0.906	0.857
DenseNet-161	16	0.800	0.817	0.885	0.900	0.923	0.865
DenseNet-169	16	0.805	0.795	0.898	0.891	0.909	0.860
DenseNet-201	16	0.805	0.812	0.891	0.886	0.916	0.862
Inception v4	16	0.796	0.832	0.899	0.917	0.934	0.876
ResNet-101	16	0.813	0.810	0.905	0.889	0.907	0.865
ResNet-152	16	0.801	0.809	0.908	0.896	0.916	0.866
ResNet-18	16	0.816	0.797	0.905	0.868	0.899	0.857
ResNet-34	16	0.799	0.798	0.902	0.891	0.905	0.859
ResNet-50	16	0.798	0.799	0.890	0.880	0.913	0.856
SqueezeNet-1.0	16	0.761	0.755	0.833	0.907	0.885	0.828
SqueezeNet-1.1	16	0.767	0.764	0.880	0.903	0.879	0.839
VGG-13	16	0.798	0.752	0.886	0.867	0.872	0.835
VGG-16	16	0.809	0.766	0.892	0.879	0.883	0.846
VGG-19	16	0.811	0.786	0.901	0.890	0.884	0.854
AlexNet	32	0.791	0.768	0.856	0.894	0.886	0.839
DenseNet-121	32	0.808	0.828	0.879	0.904	0.926	0.869
DenseNet-161	32	0.809	0.834	0.913	0.923	0.928	0.881
DenseNet-169	32	0.809	0.816	0.900	0.922	0.934	0.876
DenseNet-201	32	0.795	0.820	0.904	0.922	0.931	0.874
Inception v4	32	0.796	0.834	0.901	0.933	0.941	0.881
ResNet-101	32	0.797	0.823	0.911	0.915	0.936	0.876
ResNet-152	32	0.802	0.836	0.917	0.920	0.937	0.882
ResNet-18	32	0.796	0.822	0.908	0.903	0.911	0.868
ResNet-34	32	0.797	0.840	0.903	0.902	0.919	0.872
ResNet-50	32	0.811	0.832	0.916	0.913	0.934	0.881
SqueezeNet-1.0	32	0.773	0.769	0.880	0.913	0.895	0.846
SqueezeNet-1.1	32	0.785	0.789	0.895	0.904	0.898	0.854
VGG-13	32	0.800	0.762	0.883	0.896	0.907	0.850
VGG-16	32	0.798	0.776	0.890	0.911	0.906	0.856
VGG-19	32	0.787	0.790	0.879	0.911	0.916	0.857

This table shows the different areas under the receiver operating characteristic curve (AUROC) for each of the network architectures and individual findings as well as the pooled AUROC per model. According to the pooled AUROC, ResNet-152, ResNet-50 und DenseNet-161 were the best models, while SqueezeNet and AlexNet showed the poorest performance. For cardiomegaly, ResNet-34, ResNet-50, ResNet-152 and DenseNet-161 could surpass the CheXpert baseline provided by Irvin et al. ResnEt-50, ResNet-101, ResNet-152 and DenseNet-169 could also surpass the CheXpert baseline for pleural effusion. A batch size of 32 often lead to better results compared to a batch size of 16.

On the COVID-19 dataset, the AUROC did not substantially differ between the models with a range of the pooled values between 0.98 and 0.998 (Table 2, Appendix Figures S1–S3 in Supplementary Information). The highest pooled AUROC of 0.998 was achieved using a DenseNet-169 and DensNet-201 with AUROC values of 1.00 for the detection of COVID-19 and 0.997 for the detection of non-COVID-19 pneumonia or the absence of pneumonia.

**Table 2:** Areas Under the Receiver Operating Characteristic Curve—COVID 19 image data collection.

Network	BS	COVID-19	No pneumonia	Non-COVID-19 pneumonia	Pooled
AlexNet	16	0.986	0.977	0.977	0.980
DenseNet-121	16	1.000	0.995	0.995	0.997
DenseNet-161	16	1.000	0.996	0.996	0.997
DenseNet-169	16	1.000	0.995	0.995	0.997
DenseNet-201	16	1.000	0.996	0.995	0.997
Inception v4	16	0.995	0.985	0.985	0.988
ResNet-18	16	1.000	0.992	0.992	0.995
ResNet-34	16	1.000	0.994	0.994	0.996
ResNet-50	16	1.000	0.995	0.994	0.996
ResNet-101	16	1.000	0.995	0.994	0.996
ResNet-152	16	1.000	0.996	0.996	0.997
SqueezeNet-1.0	16	0.995	0.980	0.980	0.985
SqueezeNet-1.1	16	0.995	0.979	0.978	0.984
VGG-13	16	0.999	0.991	0.991	0.994
VGG-16	16	1.000	0.993	0.993	0.995
VGG-19	16	0.999	0.993	0.993	0.995
AlexNet	32	0.994	0.978	0.978	0.983
DenseNet-121	32	1.000	0.996	0.996	0.997
DenseNet-169	32	1.000	0.997	0.997	0.998
DenseNet-201	32	1.000	0.997	0.997	0.998
Inception v4	32	0.995	0.987	0.987	0.990
ResNet-18	32	1.000	0.993	0.993	0.995
ResNet-34	32	1.000	0.995	0.995	0.997
ResNet-50	32	1.000	0.995	0.995	0.997
ResNet-101	32	1.000	0.996	0.996	0.997
ResNet-152	32	1.000	0.995	0.995	0.997
SqueezeNet-1.0	32	0.996	0.982	0.982	0.987
SqueezeNet-1.1	32	0.998	0.981	0.980	0.986
VGG-13	32	1.000	0.992	0.992	0.995
VGG-16	32	1.000	0.995	0.995	0.997
VGG-19	32	1.000	0.995	0.995	0.997

This table shows values for the Area Under the Receiver Operating Characteristics Curve (AU-ROC). For calculation of the ROC, a one-against all approach was chosen. This means, that the models were evaluated regarding their performance in detecting one outcome (e.g. COVID-19) against the two others (e.g. no pneumonia or non-COVID-19 pneumonia). All networks achieved a very high accuracy with AUROC values greater than 0.98 for the detection of COVID-19 and values greater than 0.97 for the detection of non-COVID-19 pneumonia or absence of pneumonia.

*BS* Batchsize.

### Area Under the Precision Recall Curve and Sensitivity and Specificity

For AUPRC, CNN with less convolutional layers could achieve higher values than deeper network-architectures (Table S1 and Appendix Figures S1–S3 in Supplementary Information). The highest pooled values for the AUPRC were achieved by training VGG-16 (0.709), AlexNet (0.701) and ResNet-34 (0.688). For atelectasis, CGG-16 and AlexNet both achieved the highest AUPRC of 0.732, followed by Resnet-35 with 0.652. Cardiomegaly was most accurately detected by SqueezeNet 1.0 (0.565), Alexnet-152 (0.565) and Vgg-13 (0.563). SqueezNet 1.0 also achieved the highest AUPRC values for consolidation (0.815) followed by ResNet-152 (0.810) and ResNet-50 (0.809). The best classifications of pulmonary edema were achieved by DenseNet-169, DenseNet-161 (both 0.743) and DenseNet-201 (0.742). Finally, for pleural effusion, ResNet-101 and ResNet-152 achieved the highest AUPRC of 0.591, followed by ResNet-50 (0.590).   For an overview of sensitivities and specificities (including confidence intervals), please refer to Tables 3 and 4.

**Table 3:** Sensitivity on the CheXpert dataset.

Network	BZ	Atelectasis	Cardiomegaly	Consolidation	Edema	Effusion
AlexNet	16	0.65 (0.53–0.76)	0.65 (0.52–0.76)	0.88 (0.71–0.96)	0.86 (0.71–0.95)	0.77 (0.64–0.86)
DenseNet-121	16	0.77 (0.66–0.86)	0.65 (0.52–0.76)	0.88 (0.71–0.96)	0.83 (0.69–0.93)	0.81 (0.70–0.90)
DenseNet-161	16	0.81 (0.71–0.89)	0.70 (0.57–0.80)	0.84 (0.67–0.95)	0.88 (0.74–0.96)	0.92 (0.83–0.97)
DenseNet-169	16	0.81 (0.71–0.89)	0.86 (0.76–0.94)	0.88 (0.71–0.96)	0.81 (0.66–0.91)	0.97 (0.89–1.00)
DenseNet-201	16	0.71 (0.59–0.81)	0.74 (0.62–0.84)	0.88 (0.71–0.96)	0.83 (0.69–0.93)	0.73 (0.61–0.84)
Inception v4	16	0.72 (0.6–0.82)	0.8 (0.69–0.89)	0.84 (0.67–0.95)	0.88 (0.74–0.96)	0.81 (0.7–0.9)
ResNet-101	16	0.75 (0.63–0.84)	0.65 (0.52–0.76)	0.84 (0.67–0.95)	0.86 (0.71–0.95)	0.84 (0.73–0.92)
ResNet-152	16	0.77 (0.66–0.86)	0.73 (0.60–0.83)	0.88 (0.71–0.96)	0.86 (0.71–0.95)	0.94 (0.85–0.98)
ResNet-18	16	0.73 (0.62–0.83)	0.91 (0.81–0.97)	0.84 (0.67–0.95)	0.83 (0.69–0.93)	0.67 (0.54–0.78)
ResNet-34	16	0.76 (0.65–0.85)	0.76 (0.64–0.85)	0.84 (0.67–0.95)	0.83 (0.69–0.93)	0.97 (0.89–1.00)
ResNet-50	16	0.79 (0.68–0.87)	0.74 (0.62–0.84)	0.84 (0.67–0.95)	0.86 (0.71–0.95)	0.70 (0.58–0.81)
SqueezeNet-1.0	16	0.67 (0.55–0.77)	0.59 (0.46–0.71)	0.81 (0.64–0.93)	0.81 (0.66–0.91)	0.91 (0.81–0.96)
SqueezeNet-1.1	16	0.68 (0.56–0.78)	0.70 (0.57–0.80)	0.91 (0.75–0.98)	0.76 (0.61–0.88)	0.92 (0.83–0.97)
VGG-13	16	0.83 (0.72–0.90)	0.77 (0.65–0.87)	0.91 (0.75–0.98)	0.81 (0.66–0.91)	0.84 (0.73–0.92)
VGG-16	16	0.72 (0.60–0.82)	0.71 (0.59–0.82)	0.88 (0.71–0.96)	0.86 (0.71–0.95)	0.66 (0.53–0.77)
VGG-19	16	0.83 (0.72–0.90)	0.67 (0.54–0.78)	0.88 (0.71–0.96)	0.79 (0.63–0.90)	0.84 (0.73–0.92)
AlexNet	32	0.79 (0.68–0.87)	0.70 (0.57–0.80)	0.88 (0.71–0.96)	0.79 (0.63–0.90)	0.77 (0.64–0.86)
DenseNet-121	32	0.73 (0.62–0.83)	0.65 (0.52–0.76)	0.84 (0.67–0.95)	0.86 (0.71–0.95)	0.91 (0.81–0.96)
DenseNet-161	32	0.83 (0.72–0.90)	0.70 (0.57–0.80)	0.91 (0.75–0.98)	0.93 (0.81–0.99)	0.77 (0.64–0.86)
DenseNet-169	32	0.77 (0.66–0.86)	0.74 (0.62–0.84)	0.88 (0.71–0.96)	0.86 (0.71–0.95)	0.95 (0.87–0.99)
DenseNet-201	32	0.87 (0.77–0.93)	0.73 (0.60–0.83)	0.94 (0.79–0.99)	0.86 (0.71–0.95)	0.91 (0.81–0.96)
Inception v4	32	0.71 (0.59–0.81)	0.85 (0.74–0.92)	0.91 (0.75–0.98)	0.9 (0.77–0.97)	0.89 (0.79–0.95)
ResNet-101	32	0.76 (0.65–0.85)	0.77 (0.65–0.87)	0.81 (0.64–0.93)	0.83 (0.69–0.93)	0.78 (0.66–0.87)
ResNet-152	32	0.68 (0.56–0.78)	0.74 (0.62–0.84)	0.94 (0.79–0.99)	0.88 (0.74–0.96)	0.89 (0.79–0.95)
ResNet-18	32	0.72 (0.60–0.82)	0.76 (0.64–0.85)	0.88 (0.71–0.96)	0.79 (0.63–0.90)	0.91 (0.81–0.96)
ResNet-34	32	0.79 (0.68–0.87)	0.86 (0.76–0.94)	0.84 (0.67–0.95)	0.86 (0.71–0.95)	0.92 (0.83–0.97)
ResNet-50	32	0.84 (0.74–0.91)	0.77 (0.65–0.87)	0.84 (0.67–0.95)	0.81 (0.66–0.91)	0.88 (0.77–0.94)
SqueezeNet-1.0	32	0.59 (0.47–0.70)	0.82 (0.70–0.90)	0.84 (0.67–0.95)	0.88 (0.74–0.96)	0.72 (0.59–0.82)
SqueezeNet-1.1	32	0.75 (0.63–0.84)	0.76 (0.64–0.85)	0.91 (0.75–0.98)	0.81 (0.66–0.91)	0.94 (0.85–0.98)
VGG-13	32	0.72 (0.60–0.82)	0.74 (0.62–0.84)	0.88 (0.71–0.96)	0.83 (0.69–0.93)	0.70 (0.58–0.81)
VGG-16	32	0.80 (0.69–0.88)	0.62 (0.49–0.74)	0.84 (0.67–0.95)	0.76 (0.61–0.88)	0.91 (0.81–0.96)
VGG-19	32	0.72 (0.60–0.82)	0.70 (0.57–0.80)	0.84 (0.67–0.95)	0.88 (0.74–0.96)	0.91 (0.81–0.96)

This table gives estimates of sensitivity and specificity on the CheXpert dataset alongside their corresponding 95% confidence intervals.

**Table 4:** Specificity on the CheXpert dataset.

Network	BZ	Atelectasis	Cardiomegaly	Consolidation	Edema	Effusion
AlexNet	16	0.80 (0.71–0.86)	0.73 (0.65–0.80)	0.75 (0.68–0.82)	0.78 (0.70–0.84)	0.83 (0.76–0.89)
DenseNet-121	16	0.72 (0.64–0.80)	0.78 (0.70–0.85)	0.79 (0.72–0.85)	0.82 (0.76–0.88)	0.80 (0.72–0.86)
DenseNet-161	16	0.65 (0.56–0.74)	0.79 (0.72–0.86)	0.81 (0.74–0.87)	0.79 (0.72–0.85)	0.74 (0.66–0.81)
DenseNet-169	16	0.69 (0.60–0.76)	0.58 (0.49–0.66)	0.79 (0.73–0.85)	0.85 (0.79–0.90)	0.64 (0.56–0.72)
DenseNet-201	16	0.79 (0.71–0.85)	0.74 (0.66–0.81)	0.81 (0.74–0.87)	0.84 (0.78–0.90)	0.89 (0.83–0.94)
Inception v4	16	0.79 (0.71–0.85)	0.74 (0.66–0.81)	0.81 (0.74–0.87)	0.84 (0.78–0.9)	0.89 (0.83–0.94)
ResNet-101	16	0.77 (0.69–0.84)	0.81 (0.73–0.87)	0.83 (0.76–0.88)	0.82 (0.75–0.88)	0.78 (0.70–0.84)
ResNet-152	16	0.75 (0.66–0.82)	0.73 (0.65–0.80)	0.81 (0.74–0.87)	0.84 (0.77–0.89)	0.70 (0.62–0.78)
ResNet-18	16	0.79 (0.71–0.85)	0.51 (0.42–0.59)	0.84 (0.78–0.89)	0.81 (0.74–0.87)	0.94 (0.89–0.97)
ResNet-34	16	0.75 (0.66–0.82)	0.71 (0.63–0.79)	0.81 (0.74–0.86)	0.81 (0.74–0.87)	0.65 (0.57–0.73)
ResNet-50	16	0.69 (0.60–0.77)	0.73 (0.65–0.80)	0.77 (0.70–0.83)	0.81 (0.74–0.87)	0.93 (0.87–0.96)
SqueezeNet-1.0	16	0.72 (0.64–0.80)	0.79 (0.71–0.85)	0.77 (0.70–0.83)	0.84 (0.78–0.90)	0.69 (0.60–0.76)
SqueezeNet-1.1	16	0.72 (0.63–0.79)	0.70 (0.61–0.77)	0.75 (0.68–0.82)	0.92 (0.87–0.96)	0.65 (0.57–0.73)
VGG-13	16	0.65 (0.56–0.73)	0.60 (0.51–0.68)	0.71 (0.64–0.78)	0.81 (0.74–0.86)	0.73 (0.65–0.80)
VGG-16	16	0.78 (0.70–0.85)	0.70 (0.61–0.77)	0.78 (0.71–0.84)	0.81 (0.74–0.86)	0.93 (0.87–0.96)
VGG-19	16	0.69 (0.60–0.76)	0.76 (0.68–0.83)	0.76 (0.69–0.82)	0.86 (0.80–0.91)	0.75 (0.67–0.82)
AlexNet	32	0.68 (0.59–0.76)	0.71 (0.62–0.78)	0.72 (0.64–0.78)	0.82 (0.76–0.88)	0.81 (0.74–0.87)
DenseNet-121	32	0.76 (0.67–0.83)	0.85 (0.78–0.91)	0.79 (0.72–0.85)	0.83 (0.76–0.89)	0.78 (0.70–0.84)
DenseNet-161	32	0.65 (0.56–0.74)	0.81 (0.73–0.87)	0.83 (0.76–0.88)	0.78 (0.71–0.84)	0.91 (0.85–0.95)
DenseNet-169	32	0.72 (0.64–0.80)	0.76 (0.68–0.83)	0.82 (0.75–0.87)	0.84 (0.78–0.90)	0.72 (0.63–0.79)
DenseNet-201	32	0.61 (0.52–0.70)	0.77 (0.69–0.84)	0.76 (0.69–0.83)	0.86 (0.79–0.91)	0.77 (0.69–0.84)
Inception v4	32	0.76 (0.68–0.83)	0.64 (0.55–0.72)	0.76 (0.69–0.82)	0.85 (0.79–0.9)	0.83 (0.75–0.89)
ResNet-101	32	0.72 (0.63–0.79)	0.71 (0.63–0.79)	0.88 (0.82–0.92)	0.88 (0.81–0.92)	0.92 (0.86–0.96)
ResNet-152	32	0.80 (0.72–0.87)	0.78 (0.70–0.85)	0.75 (0.68–0.82)	0.81 (0.74–0.87)	0.83 (0.76–0.89)
ResNet-18	32	0.75 (0.66–0.82)	0.74 (0.65–0.81)	0.82 (0.76–0.88)	0.89 (0.83–0.93)	0.73 (0.65–0.80)
ResNet-34	32	0.72 (0.63–0.79)	0.65 (0.56–0.73)	0.82 (0.76–0.88)	0.84 (0.77–0.89)	0.74 (0.66–0.81)
ResNet-50	32	0.67 (0.58–0.75)	0.75 (0.67–0.82)	0.85 (0.78–0.90)	0.86 (0.80–0.91)	0.83 (0.75–0.89)
SqueezeNet-1.0	32	0.83 (0.75–0.89)	0.58 (0.49–0.66)	0.78 (0.71–0.84)	0.79 (0.72–0.85)	0.90 (0.84–0.94)
SqueezeNet-1.1	32	0.70 (0.61–0.78)	0.71 (0.62–0.78)	0.76 (0.69–0.83)	0.89 (0.83–0.93)	0.69 (0.60–0.76)
VGG-13	32	0.79 (0.71–0.85)	0.67 (0.58–0.75)	0.76 (0.69–0.82)	0.84 (0.78–0.9)	0.92 (0.86–0.96)
VGG-16	32	0.65 (0.56–0.74)	0.80 (0.72–0.86)	0.81 (0.74–0.87)	0.93 (0.88–0.97)	0.75 (0.67–0.82)
VGG-19	32	0.74 (0.65–0.81)	0.80 (0.72–0.86)	0.78 (0.71–0.84)	0.83 (0.76–0.89)	0.75 (0.67–0.82)

On the COVID-19 dataset, very high AUPRC values could be reached with all sixteen different CNN architectures for the detection of non-COVID-19 pneumonia or absence of pneumonia (Table S2 in Supplementary Information, Figs. 4, 5, 6). However, for the detection of COVID-10 pneumonia, heterogenous performances were achieved. While using Densnet-121, an AUPRC of 0.329 could be achieved, employment of a VGG-19 could achieve values of 0.925. However, it should be noted that there were only 15 COVID-19 cases in the 3,000 image test data set, so even a single misclassification likely had a major impact on the measured performance.

**Figure 4:**
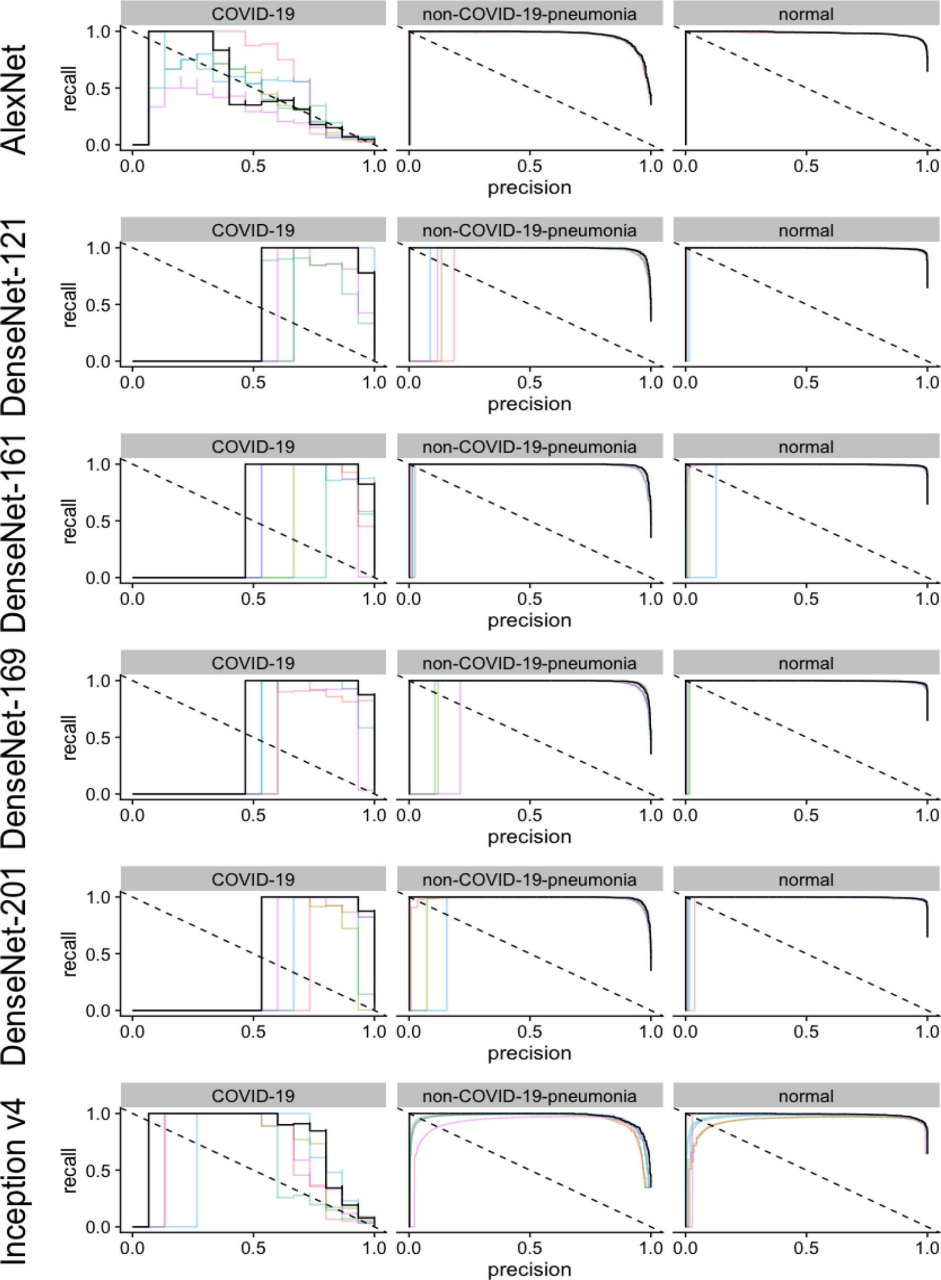
Illustration of the precision recall curves for AlexNet, DenseNet and Inception v4﻿ models. The colored lines represent a single training, black lines represent the pooled performance over five trainings. The figure was generated in R^20^.

**Figure 5:**
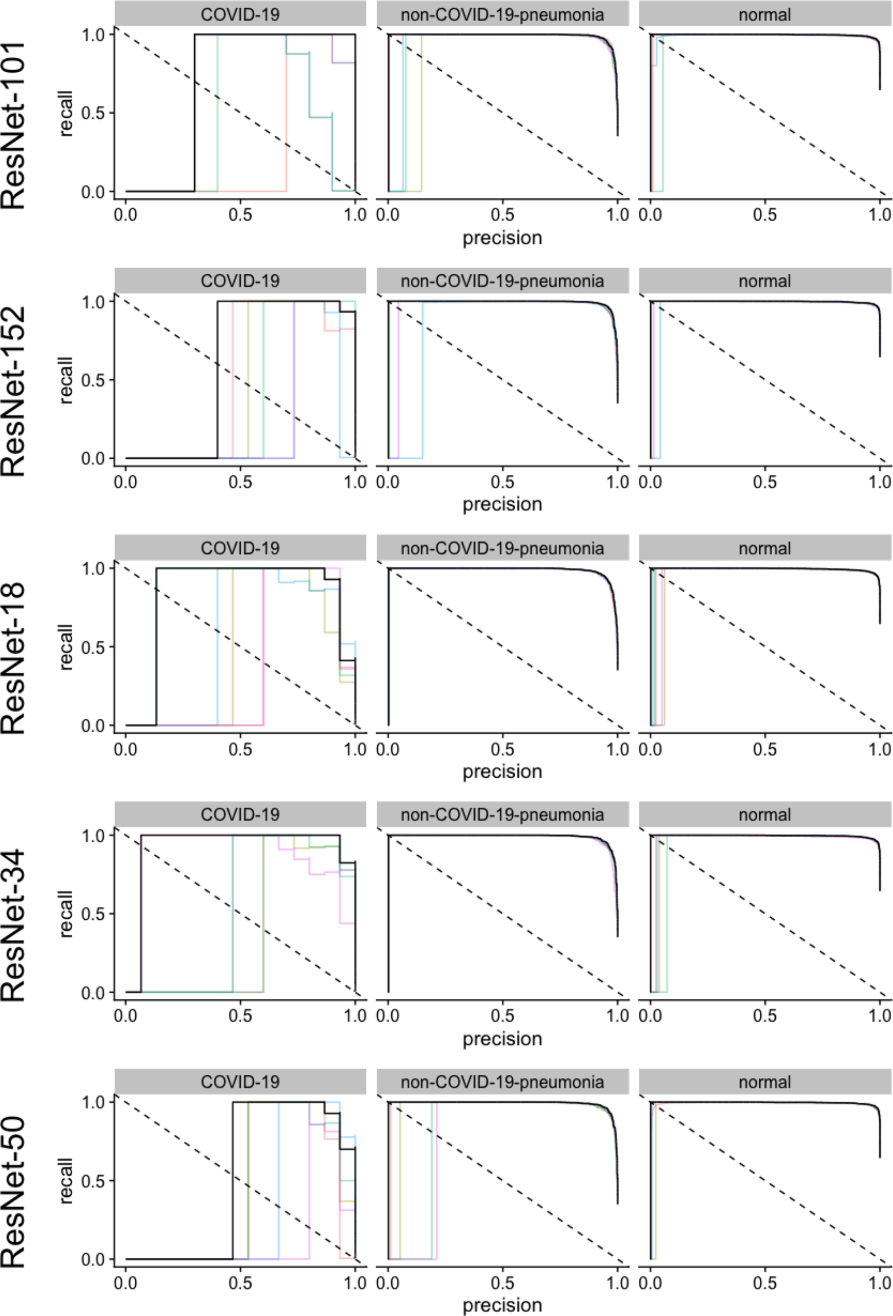
Illustration of the precision recall curves for models with ResNet architectures. The colored lines represent a single training, black lines represent the pooled performance over five trainings. The figure was generated in R^20^.

**Figure 6:**
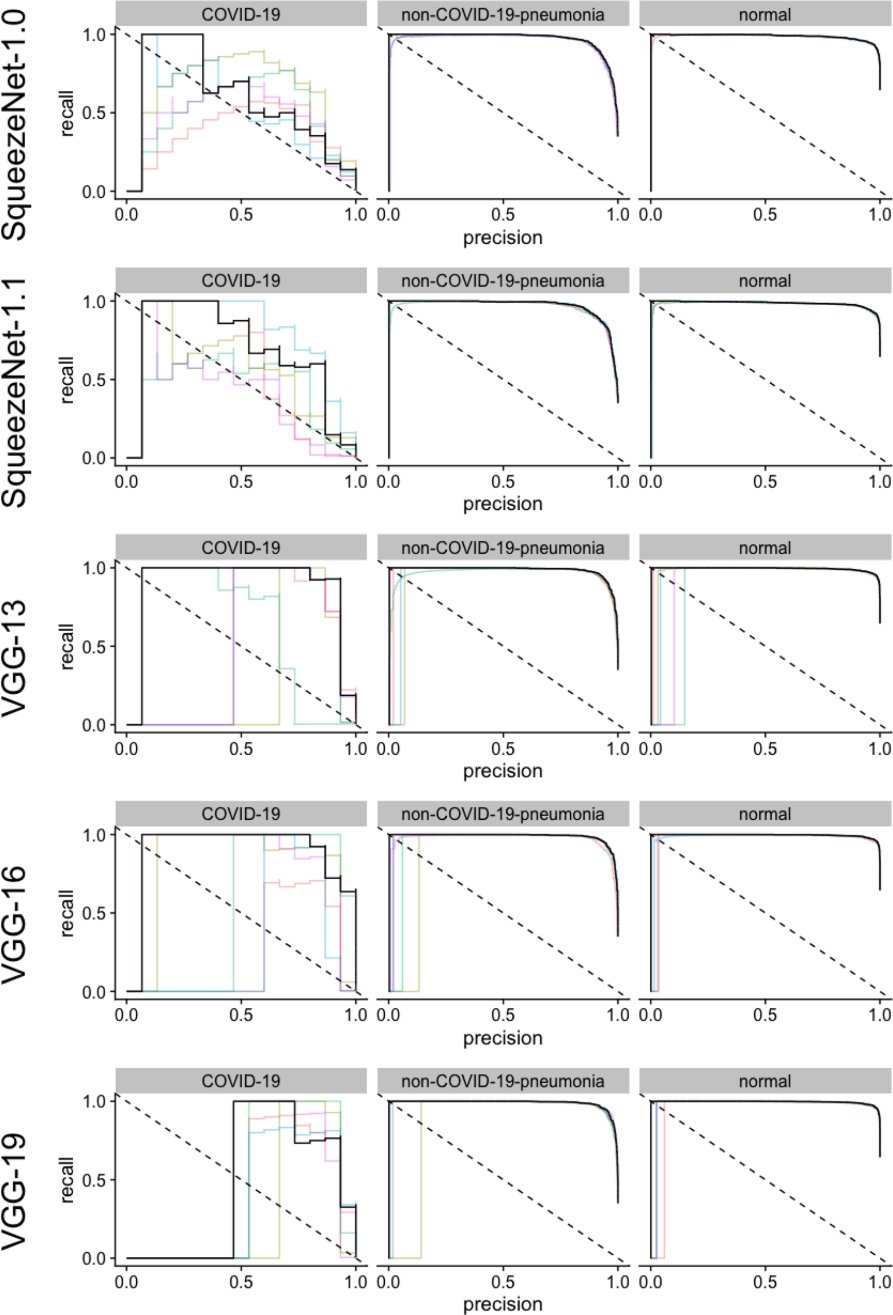
Illustration of the precision recall curves for models with Squeezenet and VGG architectures. The colored lines represent a single training, black lines represent the pooled performance over five trainings. The figure was generated in R^20^.

#### Training time

Fourteen different network-architectures were trained ten times each with a multilabel-classification head (five times each for batch size of 16 or 32 and an input-image resolution of 320 × 320 pixels) and once with a binary classification head for each finding, resulting in 210 individual training runs. Overall, the training took 340 h. As to be expected, the training of deeper networks required more time than the training of shallower networks. For an image resolution of 320 × 320 pixels, the training of AlexNet required the least amount of time with a time per epoch of 2:29–2:50 min and a total duration of 20:25 min for a batch size of 32. Using a smaller batch size of 16, the time per epoch raised to 2:59–3:06 min and a total duration of 24:01 min. In contrast, using a batch size of 16, training of a DenseNet-201 took the longest with 5:11:22 h and epochs requiring between 40:58 and 41:00 min. For a batch size of 32, training a DenseNet-169 required the largest amount of time with 3:05:49 h (epochs between 20:57 and 27:01 min). Increasing the batch size from 16 to 32 lead to an average acceleration of training by 29.9% ± 9.34%. Table 5 gives an overview of training times.

**Table 5:** Sensitivity and specificity for the detection of COVID-19 or non-COVID-19 pneumonia.

Network	BS	COVID-19 pneumonia		Non-COVID-19 pneumonia		No pneumonia	
		Sensitivity	Specificity	Sensitivity	Specificity	Sensitivity	Specificity
AlexNet	16	0.93 (0.68–1.00)	0.91 (0.90–0.92)	0.94 (0.93–0.95)	0.91 (0.89–0.93)	0.90 (0.88–0.92)	0.95 (0.94–0.96)
DenseNet-121	16	0.93 (0.68–1.00)	1.00 (1.00–1.00)	0.97 (0.96–0.98)	0.97 (0.96–0.98)	0.97 (0.96–0.98)	0.97 (0.96–0.97)
DenseNet-161	16	0.93 (0.68–1.00)	1.00 (1.00–1.00)	0.98 (0.98–0.99)	0.96 (0.95–0.97)	0.96 (0.95–0.97)	0.98 (0.98–0.99)
DenseNet-169	16	0.93 (0.68–1.00)	1.00 (1.00–1.00)	0.98 (0.97–0.98)	0.96 (0.95–0.97)	0.96 (0.94–0.97)	0.98 (0.97–0.98)
DenseNet-201	16	0.93 (0.68–1.00)	1.00 (1.00–1.00)	0.98 (0.97–0.98)	0.97 (0.95–0.98)	0.96 (0.95–0.97)	0.98 (0.97–0.98)
Inception v4	16	0.93 (0.68–1.00)	0.95 (0.94–0.95)	0.93 (0.92–0.94)	0.95 (0.94–0.96)	0.95 (0.93–0.96)	0.93 (0.92–0.94)
ResNet-18	16	0.93 (0.68–1.00)	0.99 (0.99–1.00)	0.97 (0.96–0.98)	0.95 (0.93–0.96)	0.95 (0.93–0.96)	0.97 (0.96–0.98)
ResNet-34	16	0.93 (0.68–1.00)	1.00 (1.00–1.00)	0.97 (0.96–0.98)	0.96 (0.95–0.97)	0.96 (0.94–0.97)	0.97 (0.96–0.98)
ResNet-50	16	0.93 (0.68–1.00)	1.00 (1.00–1.00)	0.98 (0.97–0.98)	0.96 (0.94–0.97)	0.96 (0.94–0.97)	0.98 (0.97–0.98)
ResNet-101	16	0.90 (0.55–1.00)	1.00 (1.00–1.00)	0.98 (0.97–0.98)	0.95 (0.94–0.96)	0.95 (0.94–0.96)	0.98 (0.97–0.98)
ResNet-152	16	0.93 (0.68–1.00)	1.00 (1.00–1.00)	0.97 (0.97–0.98)	0.96 (0.95–0.97)	0.97 (0.96–0.98)	0.97 (0.96–0.98)
SqueezeNet-1.0	16	0.93 (0.68–1.00)	0.97 (0.97–0.98)	0.94 (0.93–0.95)	0.92 (0.9–0.94)	0.90 (0.88–0.92)	0.96 (0.95–0.97)
SqueezeNet-1.1	16	0.93 (0.68–1.00)	0.95 (0.94–0.96)	0.92 (0.90–0.93)	0.94 (0.93–0.96)	0.94 (0.92–0.95)	0.92 (0.91–0.94)
VGG-13	16	0.93 (0.68–1.00)	0.98 (0.97–0.98)	0.97 (0.96–0.97)	0.94 (0.92–0.95)	0.94 (0.92–0.95)	0.97 (0.96–0.97)
VGG-16	16	0.93 (0.68–1.00)	1.00 (0.99–1.00)	0.97 (0.96–0.98)	0.95 (0.93–0.96)	0.95 (0.93–0.96)	0.97 (0.96–0.98)
VGG-19	16	0.93 (0.68–1.00)	0.99 (0.99–0.99)	0.97 (0.96–0.98)	0.95 (0.94–0.97)	0.95 (0.94–0.97)	0.97 (0.96–0.98)
AlexNet	32	0.93 (0.68–1.00)	0.95 (0.94–0.96)	0.94 (0.93–0.95)	0.92 (0.90–0.94)	0.92 (0.91–0.94)	0.94 (0.93–0.95)
DenseNet-121	32	0.93 (0.68–1.00)	1.00 (1.00–1.00)	0.98 (0.97–0.98)	0.97 (0.96–0.98)	0.97 (0.96–0.98)	0.98 (0.97–0.98)
DenseNet-161	32	0.93 (0.68–1.00)	1.00 (1.00–1.00)	0.98 (0.98–0.99)	0.98 (0.97–0.99)	0.98 (0.97–0.98)	0.98 (0.98–0.99)
DenseNet-169	32	0.93 (0.68–1.00)	1.00 (1.00–1.00)	0.99 (0.98–0.99)	0.97 (0.96–0.98)	0.97 (0.96–0.98)	0.99 (0.98–0.99)
DenseNet-201	32	0.93 (0.68–1.00)	1.00 (1.00–1.00)	0.99 (0.98–0.99)	0.98 (0.97–0.99)	0.98 (0.97–0.99)	0.99 (0.98–0.99)
Inception v4	32	0.93 (0.68–1.00)	0.94 (0.93–0.94)	0.94 (0.93–0.95)	0.95 (0.93–0.96)	0.95 (0.93–0.96)	0.94 (0.93–0.95)
ResNet-18	32	0.93 (0.68–1.00)	1.00 (1.00–1.00)	0.97 (0.96–0.97)	0.96 (0.94–0.97)	0.96 (0.94–0.97)	0.97 (0.96–0.97)
ResNet-34	32	0.93 (0.68–1.00)	1.00 (1.00–1.00)	0.99 (0.98–0.99)	0.95 (0.94–0.96)	0.95 (0.93–0.96)	0.99 (0.98–0.99)
ResNet-50	32	0.93 (0.68–1.00)	1.00 (1.00–1.00)	0.98 (0.97–0.98)	0.96 (0.95–0.97)	0.96 (0.94–0.97)	0.98 (0.97–0.99)
ResNet-101	32	0.93 (0.68–1.00)	1.00 (1.00–1.00)	0.99 (0.98–0.99)	0.96 (0.94–0.97)	0.96 (0.94–0.97)	0.99 (0.98–0.99)
ResNet-152	32	0.93 (0.68–1.00)	1.00 (1.00–1.00)	0.98 (0.97–0.98)	0.96 (0.94–0.97)	0.96 (0.94–0.97)	0.98 (0.97–0.99)
SqueezeNet-1.0	32	0.93 (0.68–1.00)	0.96 (0.95–0.96)	0.96 (0.95–0.97)	0.91 (0.89–0.93)	0.93 (0.92–0.95)	0.93 (0.92–0.95)
SqueezeNet-1.1	32	0.93 (0.68–1.00)	0.99 (0.98–0.99)	0.95 (0.94–0.96)	0.92 (0.90–0.93)	0.92 (0.91–0.94)	0.95 (0.94–0.96)
VGG-13	32	0.93 (0.68–1.00)	1.00 (0.99–1.00)	0.95 (0.94–0.96)	0.97 (0.96–0.98)	0.95 (0.94–0.97)	0.96 (0.95–0.97)
VGG-16	32	0.93 (0.68–1.00)	1.00 (1.00–1.00)	0.98 (0.97–0.98)	0.95 (0.94–0.97)	0.95 (0.94–0.96)	0.98 (0.97–0.98)
VGG-19	32	0.93 (0.68–1.00)	1.00 (1.00–1.00)	0.96 (0.95–0.97)	0.98 (0.97–0.98)	0.98 (0.96–0.98)	0.96 (0.95–0.97)

This table gives Sensitivity and specificity, as well as the corresponding confidence intervals for the COVID-19 Image Data Collection.

*BS* Batchsize.

On the COVID-19 Image Data collection, training of an epoch took between 03:52 and 11:33 min. There was not much difference in duration of an epoch between the models. This is probably primarily due to the fact that, in contrast to the CheXpert data set, in which all images are available in a resolution of 320 × 320 px, an on-the-fly down-scaling of the images to 320 × 320 px had to be performed for the COVID-19 Image Data Collection, which likely represented the performance bottleneck of the training. Considering the nevertheless very short training times, we refrained from downsizing the images to 320 × 320 px in advance (Table 6).

**Table 6:** Duration of training for the different models.

Network	Batchsize	Duration/epoch	Duration/training
AlexNet	16	2 min 59 s–3 min 6 s	24 min 1 s
DenseNet-121	16	23 min 1 s–24 min 59 s	3 h 6 min 53 s
DenseNet-161	16	28 min 59 s–36 min 1 s	4 h 17 min 20 s
DenseNet-169	16	30 min 57 s–34 min 3 s	4 h 20 min 34 s
DenseNet-201	16	40 min 58 s–41 min 0 s	5 h 11 min 22 s
Inception v4	16	25 min 43 s–27 16 s	2 h 10 min 42 s
ResNet-101	16	19 min 0 s–24 min 54 s	2 h 47 min 5 s
ResNet-152	16	27 min 1 s–27 min 57 s	4 h 6 min 46 s
ResNet-18	16	5 min 54 s–6 min 0 s	50 min 20 s
ResNet-34	16	9 min 58 s–10 min 6 s	1 h 12 min 35 s
ResNet-50	16	11 min 8 s–14 min 33 s	1 h 39 min 36 s
SqueezeNet-1.0	16	4 min 3 s–5 min 55 s	38 min 34 s
SqueezeNet-1.1	16	3 min 58 s–4 min 0 s	37 min 23 s
VGG-13	16	11 min 59 s–12 min 0 s	1 h 49 min 6 s
VGG-16	16	20 min 58 s–20 min 1 s	2 h 14 min 28 s
VGG-19	16	23 min 58 s–24 min 2 s	2 h 39 min 33 s
AlexNet	32	2 min 29 s–2 min 50 s	20 min 25 s
DenseNet-121	32	12 min 10 s–15 min 55 s	1 h 48 min 40 s
DenseNet-161	32	20 min 57 s–27 min 1 s	3 h 5 min 49 s
DenseNet-169	32	16 min 59 s–17 min 0 s	2 h 24 min 59 s
DenseNet-201	32	19 min 59 s–20 min 0 s	2 h 51 min 31 s
Inception v4	32	23 min 36 s–24 min 12 s	1 h 58 min 17 s
ResNet-101	32	13 min 59 s–14 min 0 s	2 h 7 min 47 s
ResNet-152	32	20 min 1 s–26 min 57 s	2 h 58 min 17 s
ResNet-18	32	3 min 50 s–4 min 17 s	31 min 45 s
ResNet-34	32	5 min 1 s–6 min 34 s	44 min 55 s
ResNet-50	32	8 min 18 s–8 min 28 s	1 h 15 min 54 s
SqueezeNet-1.0	32	3 min 0 s–3 min 59 s	27 min 32 s
SqueezeNet-1.1	32	2 min 57 s–3 min 0 s	24 min 42 s
VGG-13	32	9 min 41 s–14 min 9 s	1 h 30 min 32 s
VGG-16	32	16 min 55 s–17 min 0 s	1 h 46 min 32 s
VGG-19	32	12 min 59 s–13 min 0 s	2 h 1 min 42 s

This table provides an overview of training times per epoch (duration/epoch) and an overall training-time (duration/training) for each neural network. The times given are the average of five training runs.

## Discussion

In the present work, different architectures of artificial neural networks are analyzed with respect to their performance for the classification of chest radiographs. We could show that deeper neural networks do not necessarily perform better than shallow networks. Instead, an accurate classification of chest radiographs may be achieved with comparably shallow networks, such as AlexNet (8 layers), ResNet-34 or VGG-16.

The use of CNN with fewer layers has the advantage of lower hardware requirements and shorter training times compared to their deeper counterparts. Shorter training times allow to test more hyperparameters and facilitates the overall training process. Lower hardware requirements also enable the use of increased image resolutions. This could be of relevance for the evaluation of chest radiographs with a generic resolution of 2,048 × 2,048 to 4,280 × 4,280 px, where specific findings, such as small pneumothorax, require larger resolutions of input-images, because otherwise the crucial information regarding their presence could be lost due to a downscaling. Furthermore, shorter training times might simplify the integration of improvement methods into the training data, such as the implementation of ‘human in the loop’ annotations. ‘Human in the loop’ implies that the training of a network is supervised by a human expert, who may intervene and correct the network at critical steps. For example, the human expert may check the misclassifications with the highest loss for incorrect labels, thus effectively reducing label noise. With shorter training times, such feedback loops can be executed faster. In the CheXpert dataset, which was used as a groundwork for the present analysis, labels for the images were generated using a specifically developed natural language processing tool, which did not produce perfect labels. For example, the F1 scores for the mentioning and subsequent negation of cardiomegaly were 0.973 and 0.909, and the F1 score for an uncertainty label was 0.727. Therefore, it can be assumed, that there is a certain amount of noise in the training data, which might affect the accuracy of the models trained on it. Implementing a human-in-the loop approach for partially correcting the label noise could further improve performance of networks trained on the CheXpert dataset^21^. Our findings differ from applied techniques used in previous literature, where deeper network architectures, mainly a DenseNet-121, were used to classify the CheXpert data set^6,9,22^. The authors of the CheXpert dataset achieved an average overall AUROC of 0.889^3^, using a DenseNet-121, which was not surpassed by any of the models used in our analysis, although differences between the best performing networks and the CheXpert baseline were smaller than 0.01. It should be noted, however, that in our analysis the hyperparameters for the models were probably not selected as precise as in the original CheXpert paper by Irvin et al., since the focus of this work was more on the comparison of different architectures instead of the optimization of one specific network. Keeping all other hyper-parameters constant across the models might also have affected certain architectures more than others, thus lowering the comparability between the different networks we evaluated.

Also, the comparability of our approach and the CheXpert dataset might be limited as we adopted a different method for evaluation of our results, excluding lateral radiographs. Inclusion of lateral radiographs makes the dataset more diverse and maybe more challenging for different models. On the other hand, some findings such as small effusions, can only be seen on lateral radiographs and would thus be missed by our models, leading to a lower accuracy in comparison to the CheXpert baseline.

Pham et al. also used a DenseNet-121 as the basis for their model and proposed the most accurate model of the CheXpert dataset with a mean AUROC of 0.940 for the five selected findings^6^. The good results are probably due to the hierarchical structure of the classification framework, which takes into account correlations between different labels, and the application of a label-smoothing technique, which also allows the use of uncertainty labels (which were excluded in our present work). Allaouzi et al. similarly used a DenseNet-121 and created three different models for the classification of the CheXpert and ChestX-ray14, yielding an AUC of 0.72 for atelectasis, 0.87–0.88 for cardiomegaly, 0.74–0.77 for consolidation, 0.86–0.87 for edema and 0.90 for effusion^22^. Except for cardiomegaly, we achieved better values with several models (e.g. ResNet-34, ResNet-50, AlexNet, VGG-16). This suggests, that complex deep networks are not necessarily superior to more shallow networks for chest X-ray classification. At least for the CheXpert dataset, it seems that methods optimizing the handling of uncertainty labels and hierarchical structures of the data are important to improve model performance. Sabottke et al. trained a ResNet-32 for classification of chest radiographs and therefore are one of the few groups using a smaller network^9^. With an AUROC of 0.809 for atelectasis, 0.925 for cardiomegaly, 0.888 for edema and 0.859 for effusion, their network performed not as good as some of our tested networks. Raghu et al. employed a ResNet-50, an Inception-v3 as well as a custom-designed small network. Similar to our findings, they observed, that smaller networks showed a comparable performance to deeper networks^7^. Regarding the COVID-19 Image Data Collection, only few analyses have been published due to the novelty of this dataset. Nearly all tests networks performed similar, again showing that for the analysis of chest radiographs very deep and complex neuronal network architectures are not necessarily needed. Farooq and Hafeez published a model with an accuracy of 100% for the detection of COVID-19; however, they only had eight cases in their dataset and thus even less than in the present analysis^23^. The original COVID-Net achieved a sensitivity of 91% for the detection of COVID-19, which was surpassed by all models in our analysis^24^. Yet, the dataset was substantially smaller at the time of training the COVID-Net, which could have had an effect on the accuracy.

A limitation of the present work is, that only two openly available datasets were used. As a consequence, an overfitting with a lower generalizability of the results cannot be excluded and should be considered when interpreting our results. However, this is a common problem in deep learning research^25^.

## Conclusion

In the present work, we could show that increasing complexity and depth of artificial neural networks for the classification of chest radiographs is not always necessary to achieve state of the art results. In contrast to many previous studies, which mostly used a 121-layer DenseNet, we achieved comparable results with networks consisting of fewer layers (e.g. eight layers for AlexNet). Especially with limited hardware, using those networks could be advantageous because they can be trained faster and more efficiently.

## Supplementary information

Supplementary information.
