# Supplementary material for: Comparing different deep learning architectures for classification of chest radiographs
Source: Sci Rep. 2020 Aug 12;10:13590. doi: 10.1038/s41598-020-70479-z (PMC7423963; doi:10.1038/s41598-020-70479-z)

Appendix

# Tables

### **Table S1 – Area under the Precision Recall Curve - CheXpert**

| Network | Batchsize | Atelectasis | Cardiomegaly | Consolidation | Edema | Effusion | Pooled |
| --- | --- | --- | --- | --- | --- | --- | --- |
| AlexNet | 16 | 0.543 | 0.565 | 0.490 | 0.733 | 0.577 | 0.582 |
| DenseNet-121 | 16 | 0.503 | 0.554 | 0.802 | 0.733 | 0.580 | 0.634 |
| DenseNet-161 | 16 | 0.501 | 0.557 | 0.799 | 0.736 | 0.587 | 0.636 |
| DenseNet-169 | 16 | 0.500 | 0.560 | 0.805 | 0.733 | 0.582 | 0.636 |
| DenseNet-201 | 16 | 0.320 | 0.555 | 0.445 | 0.734 | 0.582 | 0.527 |
| Inception v4 | 16 | 0.501 | 0.550 | 0.804 | 0.743 | 0.589 | 0.637 |
| ResNet-101 | 16 | 0.499 | 0.558 | 0.765 | 0.735 | 0.582 | 0.628 |
| ResNet-152 | 16 | 0.503 | 0.559 | 0.808 | 0.737 | 0.584 | 0.638 |
| ResNet-18 | 16 | 0.500 | 0.559 | 0.806 | 0.727 | 0.580 | 0.634 |
| ResNet-34 | 16 | 0.506 | 0.560 | 0.804 | 0.735 | 0.580 | 0.637 |
| ResNet-50 | 16 | 0.501 | 0.557 | 0.802 | 0.733 | 0.585 | 0.636 |
| SqueezeNet-1.0 | 16 | 0.509 | 0.565 | 0.425 | 0.736 | 0.576 | 0.562 |
| SqueezeNet-1.1 | 16 | 0.505 | 0.563 | 0.400 | 0.733 | 0.575 | 0.555 |
| VGG-13 | 16 | 0.502 | 0.563 | 0.761 | 0.726 | 0.574 | 0.625 |
| VGG-16 | 16 | 0.501 | 0.559 | 0.797 | 0.733 | 0.577 | 0.633 |
| VGG-19 | 16 | 0.500 | 0.558 | 0.808 | 0.731 | 0.577 | 0.635 |
| AlexNet | 32 | 0.720 | 0.562 | 0.789 | 0.731 | 0.578 | 0.676 |
| DenseNet-121 | 32 | 0.500 | 0.558 | 0.793 | 0.736 | 0.587 | 0.635 |
| DenseNet-161 | 32 | 0.499 | 0.556 | 0.808 | 0.743 | 0.589 | 0.639 |
| DenseNet-169 | 32 | 0.499 | 0.556 | 0.805 | 0.743 | 0.588 | 0.638 |
| DenseNet-201 | 32 | 0.502 | 0.555 | 0.808 | 0.742 | 0.589 | 0.639 |
| Inception v4 | 32 | 0.507 | 0.552 | 0.492 | 0.745 | 0.593 | 0.578 |
| ResNet-101 | 32 | 0.500 | 0.558 | 0.808 | 0.740 | 0.591 | 0.639 |
| ResNet-152 | 32 | 0.502 | 0.559 | 0.810 | 0.741 | 0.591 | 0.641 |
| ResNet-18 | 32 | 0.502 | 0.557 | 0.805 | 0.736 | 0.582 | 0.636 |
| ResNet-34 | 32 | 0.652 | 0.556 | 0.806 | 0.737 | 0.585 | 0.667 |
| ResNet-50 | 32 | 0.497 | 0.555 | 0.809 | 0.740 | 0.590 | 0.638 |
| SqueezeNet-1.0 | 32 | 0.354 | 0.562 | 0.815 | 0.738 | 0.580 | 0.610 |
| SqueezeNet-1.1 | 32 | 0.506 | 0.563 | 0.804 | 0.731 | 0.577 | 0.636 |
| VGG-13 | 32 | 0.501 | 0.560 | 0.799 | 0.735 | 0.578 | 0.635 |
| VGG-16 | 32 | 0.732 | 0.561 | 0.804 | 0.739 | 0.582 | 0.684 |
| VGG-19 | 32 | 0.501 | 0.562 | 0.800 | 0.740 | 0.585 | 0.638 |

**Table S1** shows the area under the precision recall curve (AUPRC) for all networks and findings. In contrast to the AUROC, where deeper models achieved higher values, shallower networks yielded the best results for AUPRC (ResNet-24, AlexNet, VGG-16). DenseNet-201 and Squeezenet showed the lowest AUPRC values. Again, a batch size of 32 appeared to deliver better results compared to a batch size of 16.

## **Table S2 – Area under the Precision Recall Curve – COVID-19 Image Data Collection**

| Network | BS | COVID-19 | no pneumonia | non-COVID-19 pneumonia | Pooled |
| --- | --- | --- | --- | --- | --- |
| AlexNet | 16 | 0.471 | 0.985 | 0.968 | 0.808 |
| DenseNet-121 | 16 | 0.454 | 0.997 | 0.991 | 0.814 |
| DenseNet-161 | 16 | 0.522 | 0.998 | 0.993 | 0.838 |
| DenseNet-169 | 16 | 0.525 | 0.997 | 0.992 | 0.838 |
| DenseNet-201 | 16 | 0.459 | 0.998 | 0.992 | 0.816 |
| Inception v4 | 16 | 0.752 | 0.991 | 0.977 | 0.907 |
| ResNet-18 | 16 | 0.823 | 0.995 | 0.987 | 0.935 |
| ResNet-34 | 16 | 0.922 | 0.997 | 0.990 | 0.970 |
| ResNet-50 | 16 | 0.509 | 0.997 | 0.991 | 0.832 |
| ResNet-101 | 16 | 0.700 | 0.997 | 0.990 | 0.896 |
| ResNet-152 | 16 | 0.596 | 0.997 | 0.992 | 0.862 |
| SqueezeNet-1.0 | 16 | 0.572 | 0.987 | 0.971 | 0.843 |
| SqueezeNet-1.1 | 16 | 0.695 | 0.986 | 0.969 | 0.883 |
| VGG-13 | 16 | 0.870 | 0.993 | 0.984 | 0.949 |
| VGG-16 | 16 | 0.886 | 0.996 | 0.987 | 0.956 |
| VGG-19 | 16 | 0.439 | 0.996 | 0.988 | 0.808 |
| AlexNet | 32 | 0.643 | 0.985 | 0.969 | 0.866 |
| DenseNet-121 | 32 | 0.329 | 0.997 | 0.988 | 0.771 |
| DenseNet-169 | 32 | 0.459 | 0.998 | 0.993 | 0.817 |
| DenseNet-201 | 32 | 0.400 | 0.997 | 0.995 | 0.797 |
| Inception v4 | 32 | 0.752 | 0.992 | 0.980 | 0.908 |
| ResNet-18 | 32 | 0.522 | 0.985 | 0.989 | 0.832 |
| ResNet-34 | 32 | 0.663 | 0.997 | 0.989 | 0.883 |
| ResNet-50 | 32 | 0.922 | 0.997 | 0.991 | 0.970 |
| ResNet-101 | 32 | 0.467 | 0.997 | 0.992 | 0.819 |
| ResNet-152 | 32 | 0.859 | 0.997 | 0.992 | 0.949 |
| SqueezeNet-1.0 | 32 | 0.763 | 0.988 | 0.973 | 0.908 |
| SqueezeNet-1.1 | 32 | 0.763 | 0.988 | 0.972 | 0.908 |
| VGG-13 | 32 | 0.905 | 0.995 | 0.986 | 0.962 |
| VGG-16 | 32 | 0.592 | 0.994 | 0.989 | 0.858 |
| VGG-19 | 32 | 0.925 | 0.997 | 0.994 | 0.972 |

Table S2 shows areas under the Precision Recall Curves (AUPRC). Similar to the receiver operating characteristics analysis a one-against all approach was also chosen for calculating the precision recall curves. While high values for the detection of non-COVID-19 pneumonia or normal chest radiographs could be achieved, the AUPRC were heterogeneous for the detection of COVID-19. Highest AUPRC value for detection of COVID-19 was achieved with a ResNet-50, while the lowest values were achieved using a DenseNet-121. BS = Batchsize

# Figures

## **Receiver Operating Characteristic Curves – COVID-19 Image Data Collection**

Figures 1, 2 and 3 display the ROC-curves for all models. The colored lines represent a single training, black lines represent the pooled performance over five trainings.

### **Figure S1**


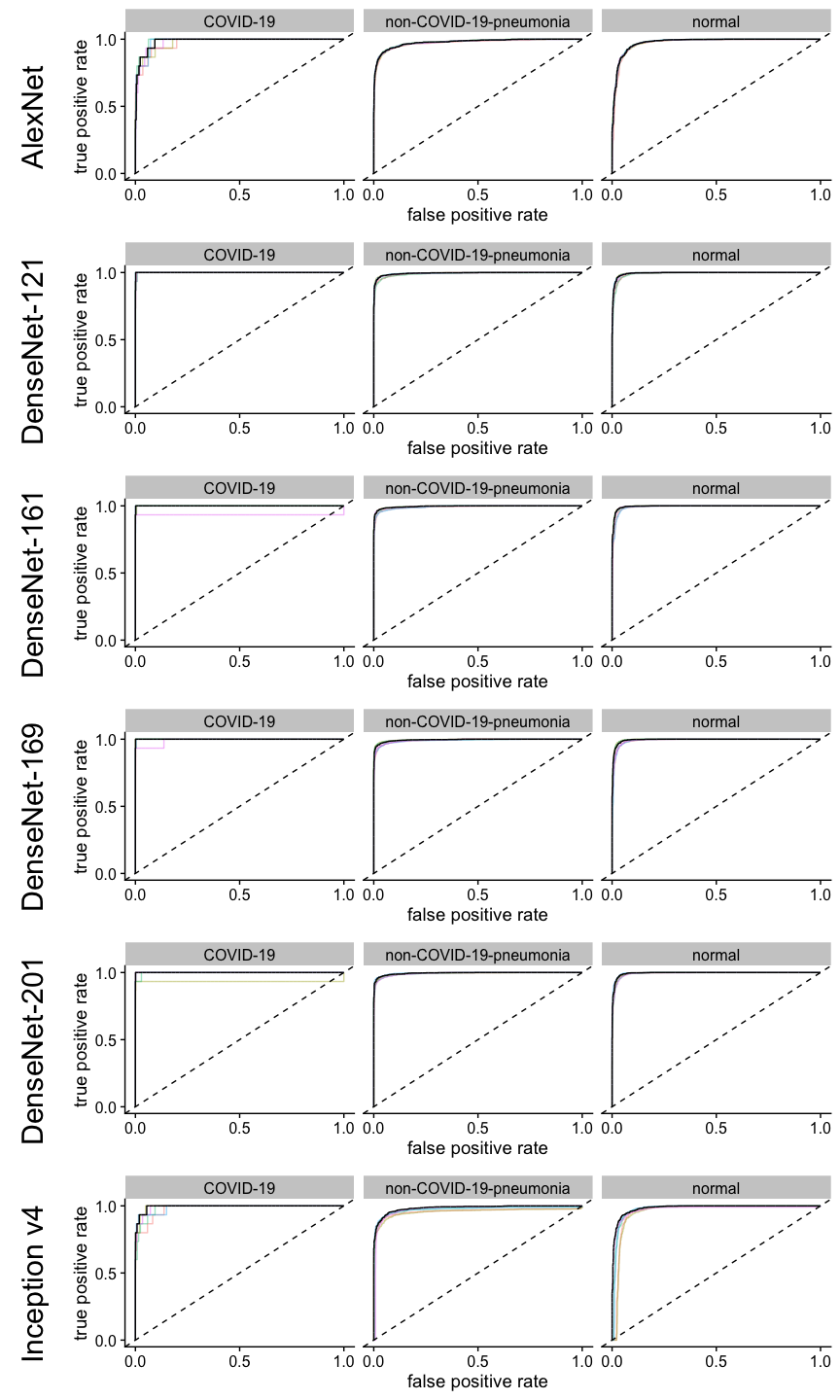


### Figure S2


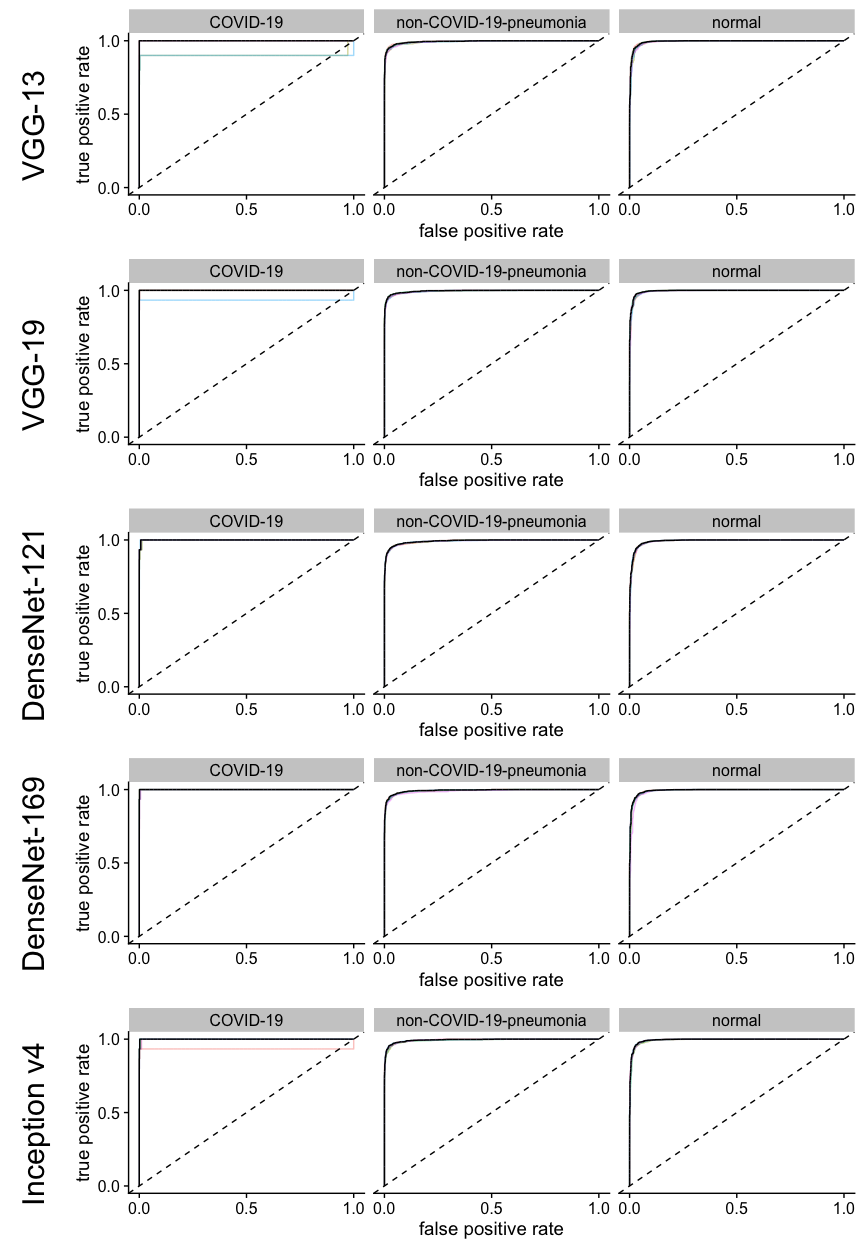


### Figure S3


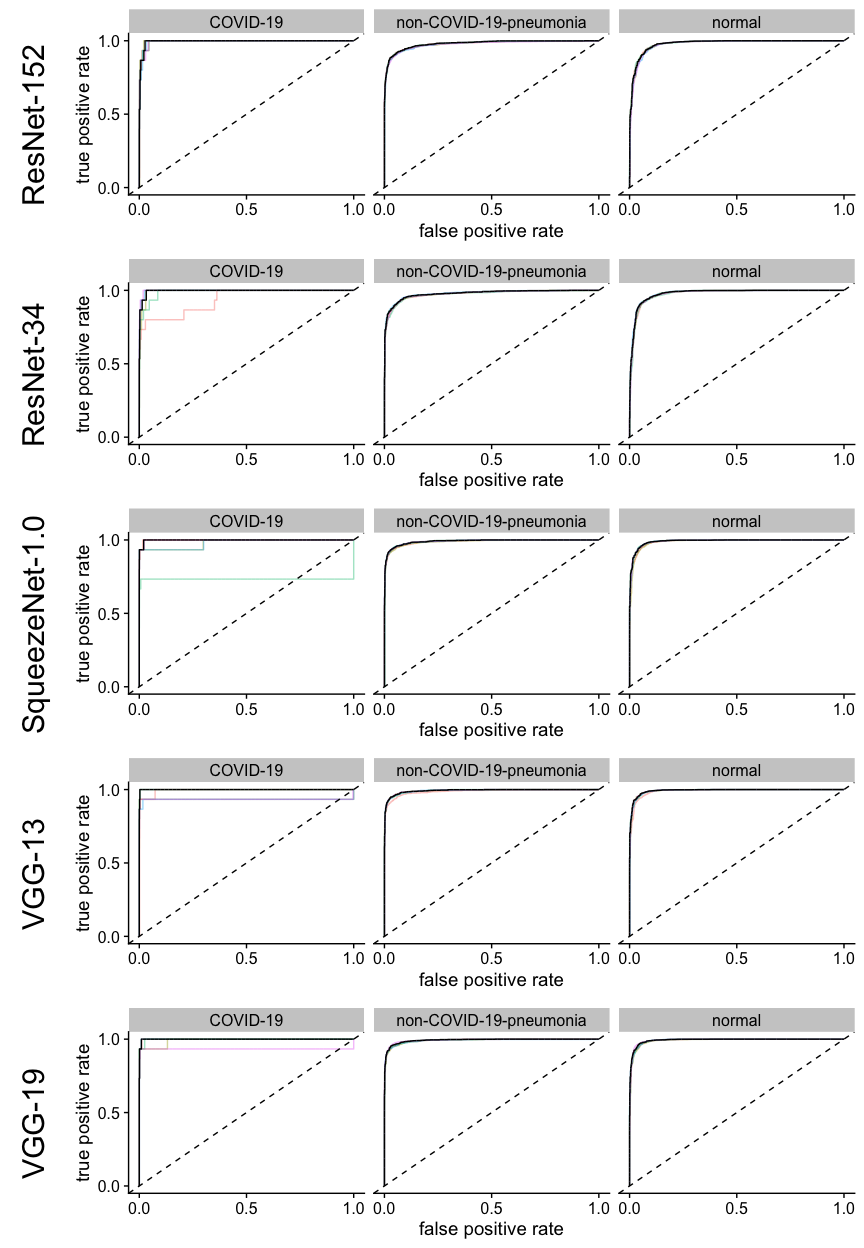


## Precision Recall Curves - CheXpert

### Figure S4


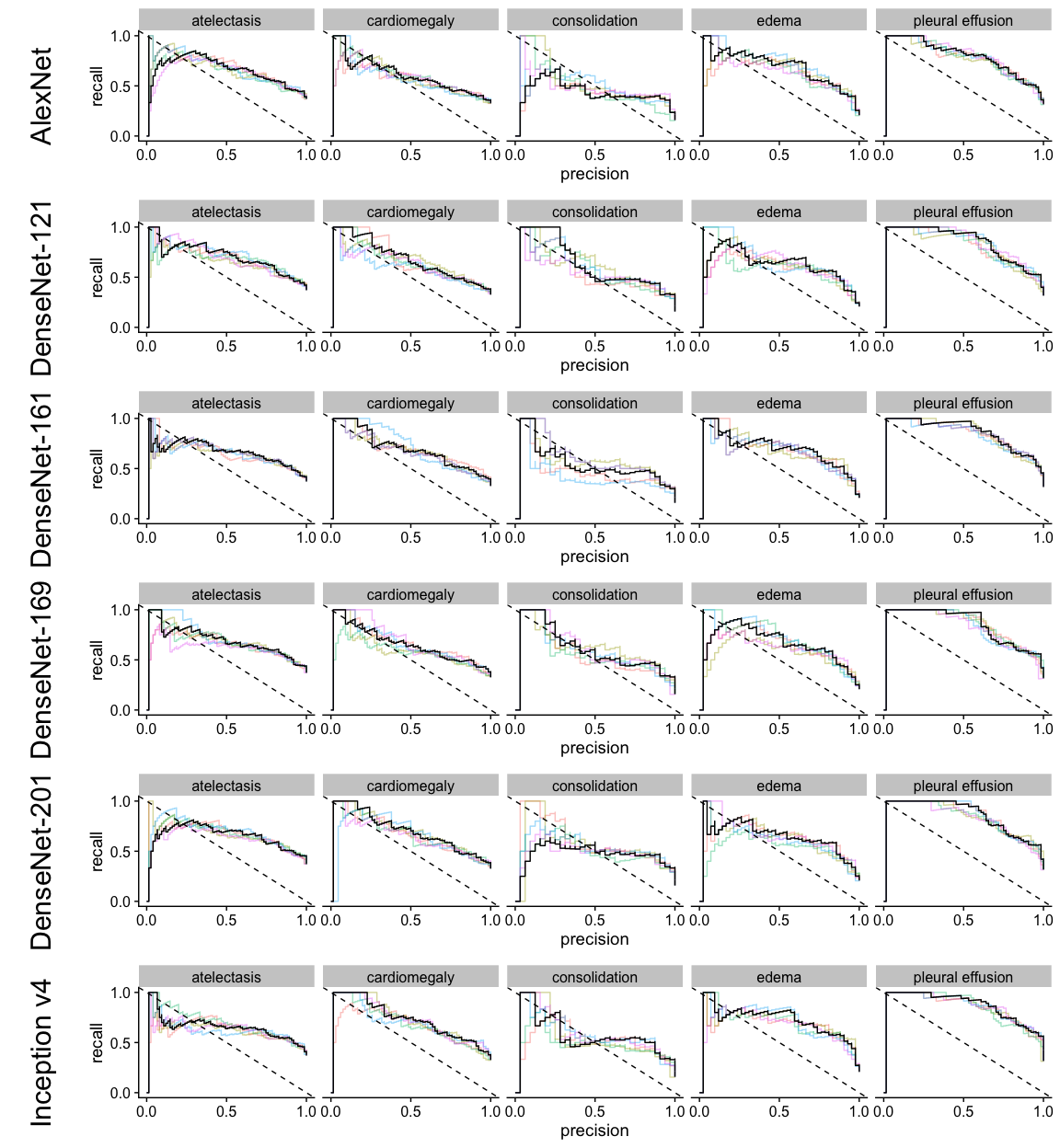


### Figure S5


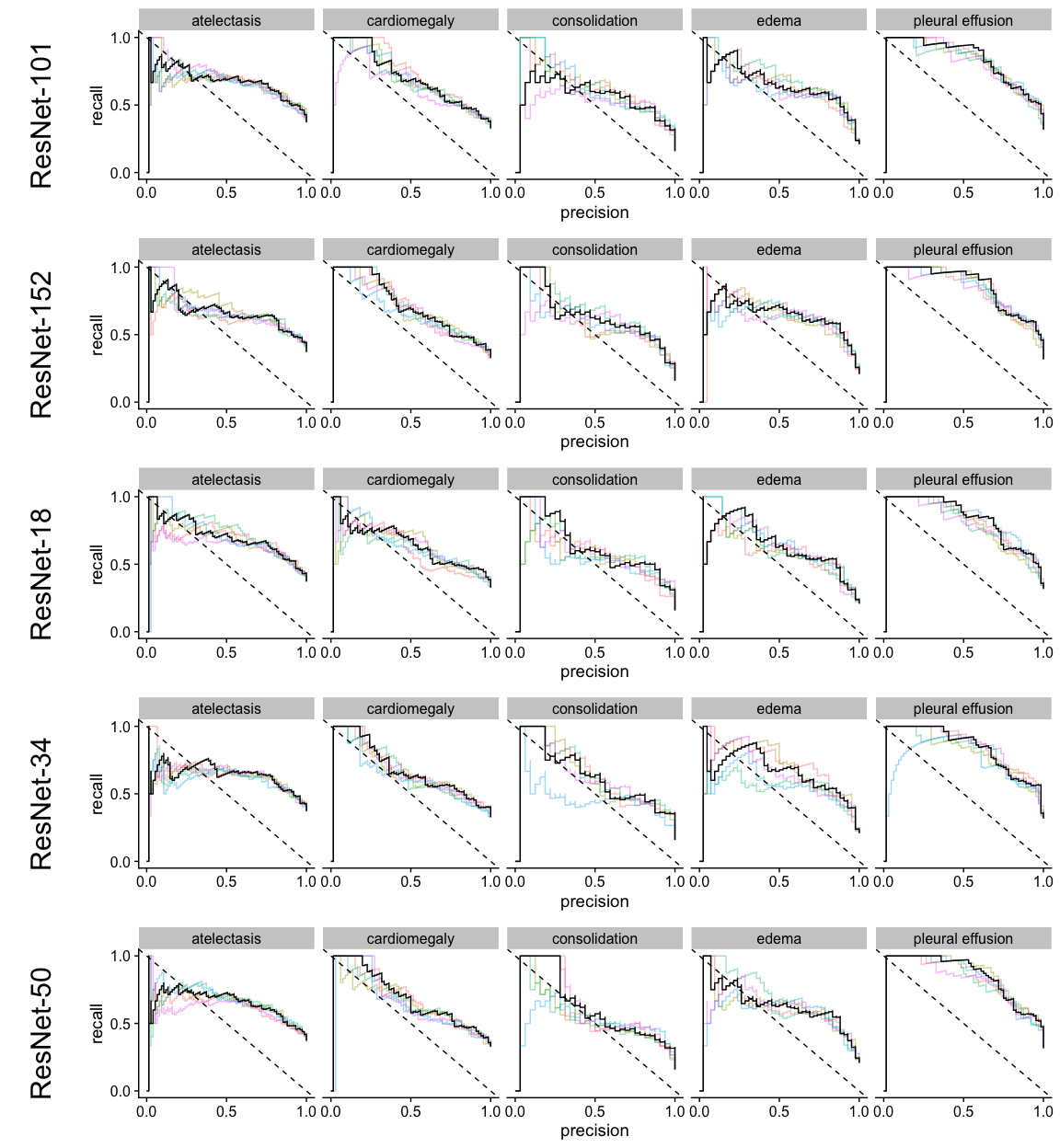


### Figure S6


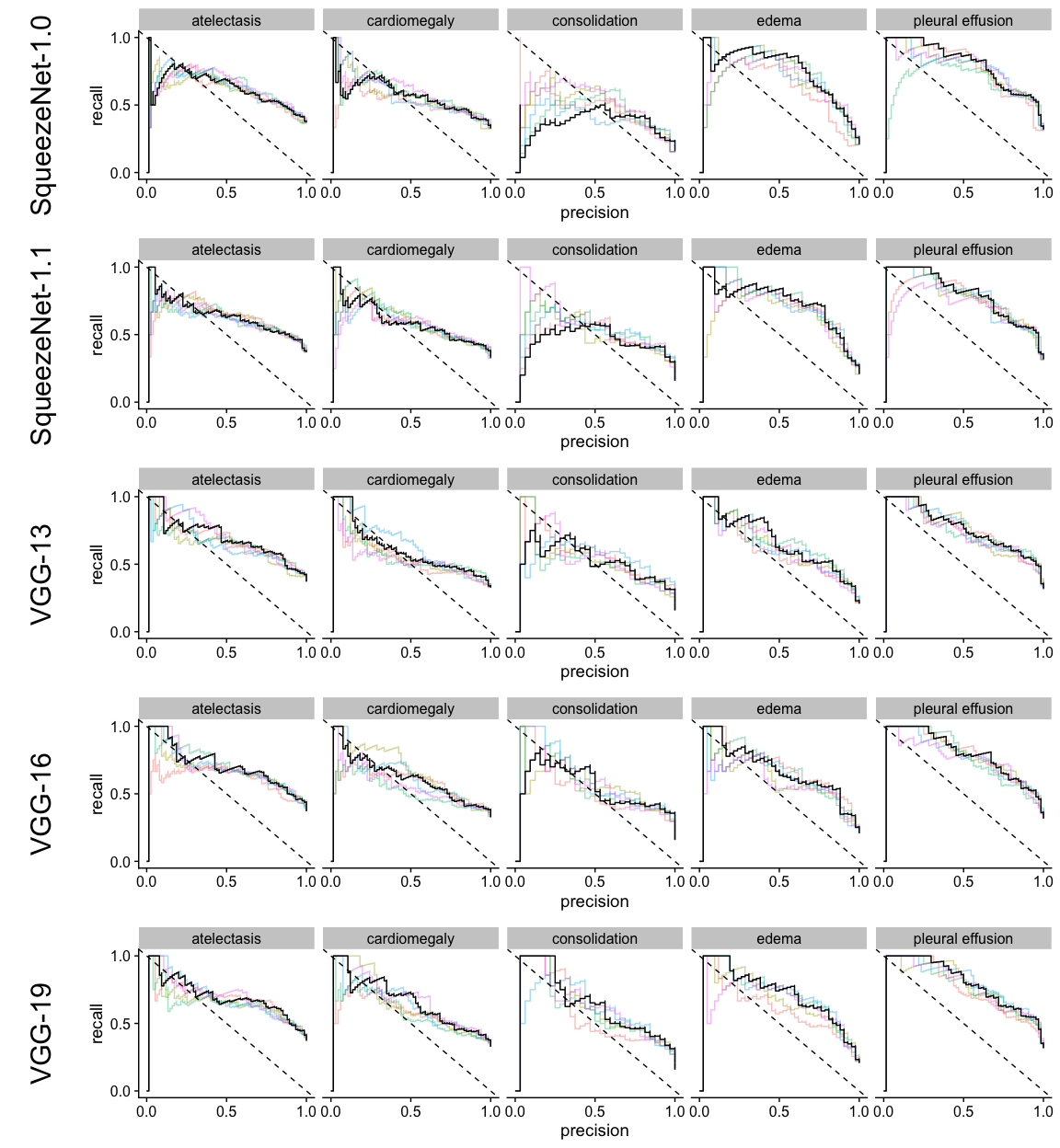

Supplement: Supplementary file 1 — Supplementary information. [file 41598_2020_70479_MOESM1_ESM.docx]
